# Predicting the risk of chest radiograph abnormality 12-weeks post hospitalisation with SARS CoV-2 PCR confirmed COVID-19

**DOI:** 10.1186/s12931-022-02217-0

**Published:** 2022-10-31

**Authors:** Tim JM Wallis, Benjamin Welham, Alex Kong, Tommaso Morelli, Adnan Azim, Jose Horno, Miranda Wilkinson, Hannah Burke, Anna Freeman, Thomas MA Wilkinson, Mark G Jones, Benjamin G Marshall

**Affiliations:** 1 Department of Respiratory Medicine and Southampton NIHR Biomedical Research Centre, School of Clinical and Experimental Sciences, Faculty of Medicine, University Hospital Southampton, University of Southampton, Southampton, UK; 2 Department of Respiratory Medicine, University Hospital Southampton, Southampton, UK

**Keywords:** COVID-19, SARS-CoV-2, Chest radiograph, LDH

## Abstract

**Background:**

Routine follow-up of patients hospitalised with COVID-19 is recommended, however due to the ongoing high number of infections this is not without significant health resource and economic burden. In a previous study we investigated the prevalence of, and risk factors for, persistent chest radiograph (CXR) abnormalities post-hospitalisation with COVID-19 and identified a 5-point composite score that strongly predicted risk of persistent CXR abnormality at 12-weeks. Here we sought to validate and refine our findings in an independent cohort of patients.

**Methodology:**

A single-centre prospective study of consecutive patients attending a virtual post-hospitalisation COVID-19 clinic and CXR as part of their standard clinical care between 2nd March – 22nd June 2021. Inpatient and follow-up CXRs were scored by the assessing clinician for extent of pulmonary infiltrates (0–4 in each lung) with complete resolution defined as a follow-up score of zero.

**Results:**

182 consecutive patients were identified of which 31% had persistent CXR abnormality at 12-weeks. Patients with persistent CXR abnormality were significantly older (p < 0.001), had a longer hospital length of stay (p = 0.005), and had a higher incidence of both level 2 or 3 facility admission (level 2/3 care) (p = 0.003) and ever-smoking history (p = 0.038). Testing our composite score in the present cohort we found it predicted persistent CXR abnormality with reasonable accuracy (area under the receiver operator curve [AUROC 0.64]). Refining this score replacing obesity with Age ≥ 50 years, we identify the *SHADE-750 score* (1-point each for; Smoking history, Higher-level care (level 2/3 admission), Age ≥ 50 years, Duration of admission ≥ 15 days and Enzyme-lactate dehydrogenase (LDH ≥ 750U/L), that accurately predicted risk of persistent CXR abnormality, both in the present cohort (AUROC 0.73) and when retrospectively applied to our 1st cohort (AUROC 0.79). Applied to both cohorts combined (n = 213) it again performed strongly (AUROC 0.75) with all patients with a score of zero (n = 18) having complete CXR resolution at 12-weeks.

**Conclusions:**

In two independent cohorts of patients hospitalised with COVID-19, we identify a 5-point score which accurately predicts patients at risk of persistent CXR abnormality at 12-weeks. This tool could be used by clinicians to identify patients in which radiological follow-up may not be required.

**Supplementary Information:**

The online version contains supplementary material available at 10.1186/s12931-022-02217-0.

## Background

Despite novel COVID-19 treatments and vaccines, the COVID-19 pandemic continues with high levels of infection and there is now appreciation in the scientific community that the COVID-19 pandemic will end with the severe acute respiratory syndrome coronavirus – 2 (SARS CoV-2) virus becoming endemic [1]. National and international guidelines have been published outlining follow-up strategies for patients hospitalised with COVID-19 [2, 3]. Current British Thoracic Society (BTS) guidance recommends a follow-up chest radiograph (CXR) at 12-weeks for all patients hospitalised with COVID-19 pneumonia [3] to monitor for potential long term sequelae such as post-COVID fibrosis [4]. Since implementation of these guidelines, studies have identified persistent CXR abnormality in 13–38% of COVID-19 survivors at 2–3 month follow-up [5–7].

As a consequence of the ongoing high-rate of infection the follow-up of patients hospitalised with COVID-19 has a significant and ongoing health resource and economic burden [8, 9]. Hence there is clinical need for the development of prognostic tools which aid triage of patients into those who require routine follow-up and those where it may not be necessary.

In a previous article, [7] we described outcomes of a prospective study of patients hospitalised during the U.K 1st wave of the COVID-19 pandemic (March-June 2020) attending virtual follow-up. In this article 32% of patients had persistent CXR abnormality at 12-weeks and using a five-point composite model (1-point each for; hospital length of stay ≥ 15 days, Level 2 or 3 care facility admission, admission lactate dehydrogenase [LDH] ≥ 750 U/L, obesity, and ever smoking-status) strongly predicted patients at risk of persistent abnormality (area under the receiver operating characteristic curve [AUROC] 0.81).

In this study we sought to validate and refine the risk score in an independently recruited second cohort of patients. With the aim of testing how accurately it could identify a group of patients who do not require routine CXR follow up following hospitalisation with SARS CoV-2 infection.

## Methods

This was a prospective cohort study at a single academic medical centre, University Hospital Southampton NHS Foundation Trust (UHSFT). The present cohort comprised consecutive patients hospitalised with PCR confirmed symptomatic SARS CoV-2 infection attending for a 12-week virtual follow-up clinic and CXR as part of their standard clinical care between 2nd March – 22nd June 2021. Ethical approval was obtained from the South-Central Hampshire A Research Ethics Committee (REC reference (20/SC/0138) as part of the REACT study (REal-time Analytics for Clinical Trials observational study of COVID-19). Informed consent was waived because of the study design.

The general methodology for the study is as previously described [7]. Briefly, baseline and 12-week follow-up CXRs were scored for COVID-19 related pulmonary infiltrates by the assessing clinician at the virtual follow-up appointment from 0 to 4 for each lung (0 = nil, 1 = < 25%, 2 = 25–50%, 3 = 51–75%, 4 = > 75%) [10]. The baseline CXRs were defined as the last film prior to patient’s discharge (or the admission film if only one radiograph was performed). This was either a posteroanterior (PA) or anteroposterior (AP) film as performed during standard of care. Follow-up outpatient films were all departmental PA chest radiographs.

Level 2 care was defined as patients requiring single organ support excluding mechanical ventilation (High dependency unit) and level 3 care those requiring mechanical ventilation alone or two or more organ support (Intensive care unit).

Baseline and 12-week follow-up LDH samples were taken as part of routine standard clinical care and analysed at UHSFT. Here, continuous data is presented as the median and interquartile range (IQR) with comparisons between data made using the Mann Whitney U-test and Chi-squared test (χ2) as appropriate. Association between continuous variables was assessed using Spearman’s correlation coefficient (r). Model discrimination is presented using the area under the receiver operating characteristic curve (AUROC). We first tested the performance of our previously published 5-point composite score. This score was then revised and re-tested with the aim of increasing its performance based on associated risk factors identified in the present study. P values of < 0.05 were deemed significant. Statistical analysis was conducted using IBM®-SPSS® (version 26).

## Results

One-hundred and eighty-two (n = 182) consecutive patients were identified 54% of which were male. The median (IQR) hospital length of stay was 9 days [8–13] and median CXR follow-up interval was 80 days (74–96) (Table 1). Compared to our previous study, patients in the present study were non-significantly older (median 58 years vs. 54 years p = 0.07), had a higher incidence of obesity (40% vs. 28% p = 0.03) and a lower incidence of hypertension (23% vs. 35% p = 0.03). Furthermore, a lower proportion of patients had a level 2/3 care facility admission (31% vs. 49% p < 0.01), this reflecting the enrichment of our previous study cohort for patients requiring higher level care (**Supplemental Table **1).

**Table 1:** Baseline characteristics for present study cohort (n = 182). Comparing those with complete chest radiograph resolution (n = 126) versus those with persistent abnormality (n = 56). Values presented as median (Interquartile range) for continuous variables and percentage (n) for categorical variables. CXR-chest radiograph, Level 2 - High Dependency Facility, Level 3-Intensive Care Facility, IMV- Invasive mechanical ventilation, CPAP-continuous positive pressure ventilation, NIV – non-invasive ventilation, BAME -Black, Asian and Minority Ethnic. BMI body mass index (kg/m^2^), LDH- serum lactate dehydrogenase ^data available for baseline LDH n = 165 and follow-up LDH n = 172. *p < 0.05 **p < 0.01. Comparison of data for complete resolution versus persistent CXR abnormality assessed using the Mann-Whitney U Test or Chi Squared test as appropriate

Variable	Whole Group (n = 182)	Complete Resolution (n = 126)	Persistent CXR Abnormality (n = 56)	p value
**Sex (male)**	54% (n = 98)	51% (n = 64)	61% (n = 34)	0.235
**Age (years)**	58.0 (47–67)	55.0 (45–64)	64.0 (54–74)	< 0.001**
**Hospital length of Stay (days)**	9.0 (8–13)	7.0 (4–12)	10.5 (7–16)	0.005**
**Follow-up CXR interval (days)**	80.0 (74–86)	81.0 (75–86)	79.0 (72–85)	0.144
**Level 2 or 3 Care**	31% (n = 57)	25% (n = 31)	46% (n = 26)	0.003**
**IMV**	8% (n = 15)	8% (n = 10)	9% (n = 5)	0.822
**CPAP/NIV**	24% (n = 43)	16% (n = 20)	41% (n = 23)	< 0.001**
**Oxygen or higher resp. support**	92% (n = 167)	90% (n = 114)	95% (n = 53)	0.345
**BAME**	27% (n = 47)	29% (n = 35)	23% (n = 12)	0.407
**Obesity (BMI > 30 kg/m** ^**2**^ **)**	40% (n = 72)	42% (n = 53)	34% (n = 19)	0.300
**Ever smoker**	44% (n = 80)	39% (n = 49)	55% (n = 31)	0.038*
**Hypertension**	23% (n = 42)	25% (n = 31)	20% (n = 11)	0.464
**Diabetes Melitus (all types)**	20% (n = 37)	22% (n = 28)	16% (n = 9)	0.341
**Baseline LDH U/L^**	643 (506–854)	626 (501–830)	667 (535–959)	0.240
**Follow-up LDH U/L^**	387 (348–449)	379 (329–443)	407 (367–456)	0.032*

At the 12-week virtual follow-up, the most common persistent symptoms in the present study were dyspnoea in 39% and fatigue in 35%. 73% (73%) of patients were discharged following their 12-week virtual appointment.

The median CXR scores at baseline and 12-week follow-up were 5.0 [3–6] and 0.0 (0–1) respectively. Persistent CXR abnormality (score ≥ 1) was present in 31% (n = 56) of the cohort at 12-weeks, in whom 30% (n = 17) had an infiltrate score of 1, 39% (n = 22) a score of 2, and 30% (n = 17) a score of 3 or more (**Supplemental Fig. **1).

Compared to those with complete resolution, patients with persistent CXR abnormality were significantly older (64 years vs. 55 years p < 0.001), had longer hospital length of stay (10.5 days vs. 7.0 days p < 0.01), and had a higher incidence of both level 2/3 care facility admission (46% vs. 25% p < 0.01) and ever-smoking history (55% vs. 39% p = 0.04). There was no statistical difference between patients with persistent abnormality and complete resolution for requirement for supplemental oxygen, obesity, or CXR follow-up interval.

No significant between group difference was observed in baseline LDH (667 U/L vs. 626 U/L p = 0.24) (Table 1), or significant correlation observed between baseline LDH and follow-up CXR score (r = 0.06 p = 0.49). However, follow-up LDH was significantly higher in patients with persistent CXR abnormality (406 U/L vs. 379 U/L p = 0.03). In addition, baseline LDH was significantly positively correlated with baseline CXR scores (r = 0.23 p < 0.01) and follow-up LDH concentrations (r = 0.33 p < 0.001), whereas follow-up LDH was significantly correlated with both baseline (r = 0.26 p < 0.01) and follow-up CXR score (r = 0.20 p < 0.01).

**Fig. 1:**
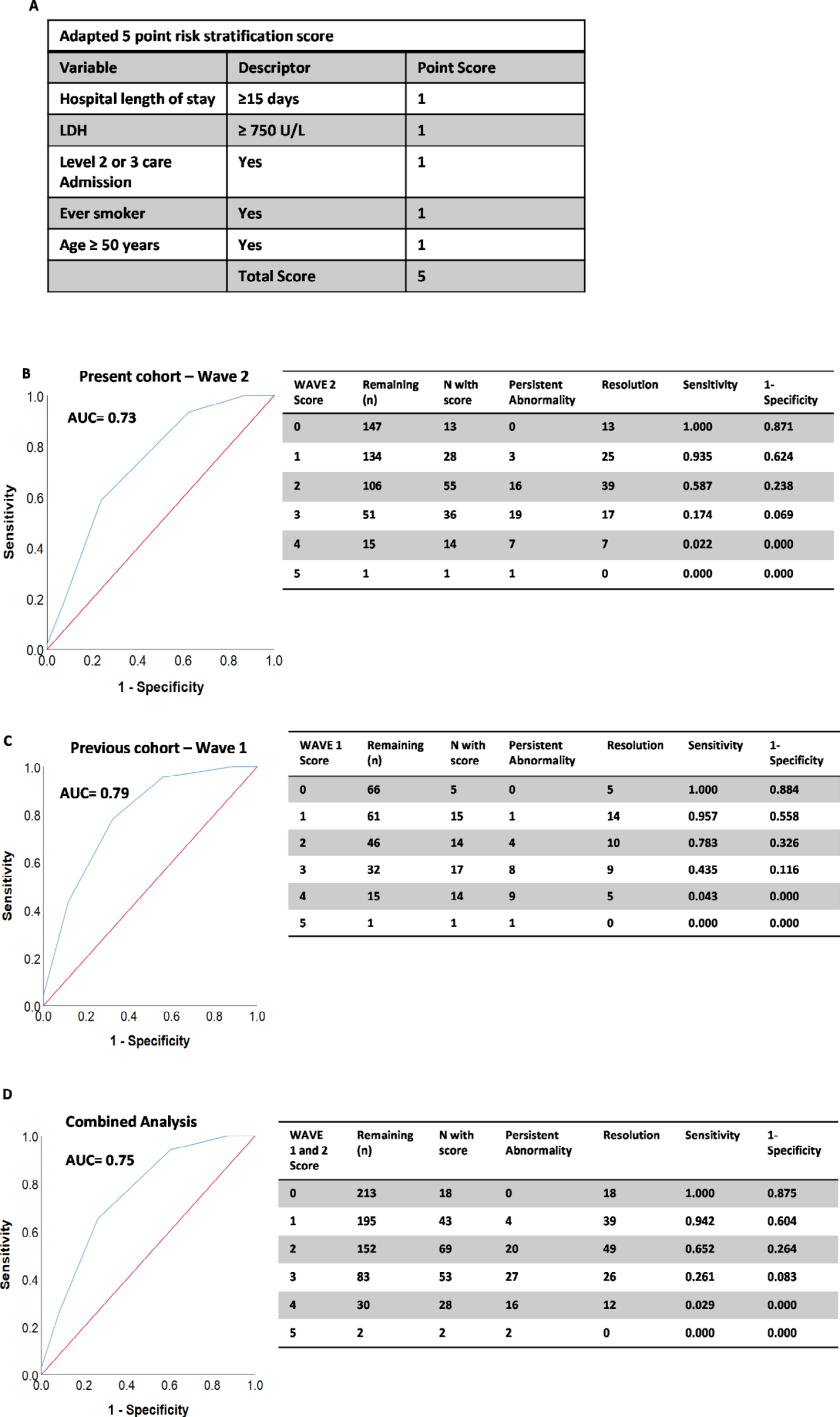
**Area under receiver operating curve for risk of persistent chest radiograph abnormality using the ‘*SHADE-750’*5-point risk stratification score.** (A) ***SHADE-750 score*** (1-point each for; ***S***moking history (Ever vs. Never), ***H***igher-level care (level 2 or 3 care admission), ***A***ge ≥ 50 years, ***D***uration of admission ≥ 15 days and ***E***nzyme-lactate dehydrogenase [LDH] ≥ 750U/L). (B) Present study cohort (2nd Wave cohort) n = 147 with available data for score, of which persistent CXR abnormality n = 46 and complete resolution n = 101, (C) Previous study 1st Wave cohort (Wave 1) n = 66 with available data for score, of which persistent CXR abnormality n = 23 and complete resolution n = 43, (D) Present study and previous study cohorts combined (Wave 1 and 2) n = 213 with available data for score, of which persistent CXR abnormality n = 69 and complete resolution n = 144

Testing our previously published 5-point score, we found it predicted patients at risk of persistent CXR abnormality with reasonable accuracy in this cohort, AUROC 0.64 (95% confidence interval [CI] 0.54–0.72) p = 0.047. In the present cohort obesity was not associated with persistent CXR abnormality and this factor detracted from the score performance. As age is a key risk factor for disease severity in COVID-19 [11], and in the present study associated with persistent CXR abnormality, in our present study we tested an adapted 5-point score replacing obesity with patient age. Using a stratification of age ≥ 50 years we developed the ***SHADE-750 score*** (1-point each for; ***S***moking history, ***H***igher-level care (level 2/3 admission), ***A***ge ≥ 50 years, ***D***uration of admission ≥ 15 days and ***E***nzyme-LDH ≥ ***750*** U/L) (Fig. 1a). This adapted 5-point score accurately predicted risk of persistent abnormality in this cohort, AUROC 0.73 (95%CI 0.65–0.82) p < 0.0001 (Fig. 1b). Retrospective application of this adapted score to our 1st wave cohort identified that it also had strong discrimination, AUROC 0.79 (95%CI 0.68–0.90) p < 0.0001 (Fig. 1c). When applying the adapted model to both cohorts combined (n = 213) it again performed strongly (AUROC 0.75 [95%CI 0.69–0.82] p < 0.0001) (Fig. 1d). Furthermore, in this combined analysis, persistent CXR abnormality was not identified in any patients with a combined score of 0 (n = 18 with score, negative predictive value [NPV] 100%), and was present in only 4 patients with a score of 1 (n = 43 with score, NPV of score 0–1 94%).

## Discussion

In this prospective cohort study, we confirm previous observations that persistent CXR abnormality is present in approximately 30% of patients hospitalised with COVID-19 at 12-week follow-up. Furthermore, in two independent cohorts of patients we identify a 5-point risk stratification tool, the ***SHADE-750*** score, that accurately predicted patients at risk of persistent CXR abnormality at 12-weeks. In combined analysis (n = 213), persistent CXR abnormality was not identified in any patients with a combined score of zero and we propose that this score could be used to accurately identify a group of patients for which routine radiological follow-up is not required.

The rate of persistent CXR abnormality identified in the present study is similar to that observed in previous reports.[5–7] Our finding that hospital length-of-stay and higher-level respiratory support are associated with persistent radiological abnormality at the 3 month follow-up timepoint is consistent with results of prospective studies using computed-tomography (CT) scanning [12, 13]. Our observation that age and smoking are associated with persistent CXR abnormality is consistent with both factors being associated with increased risk of greater COVID-19 disease severity [11, 14].

LDH is a non-specific marker of tissue inflammation and elevated LDH is associated with disease severity [15] and acute respiratory distress syndrome in COVID-19 [16]. In our previous study we observed significantly higher baseline LDH concentrations in patients with persistent CXR abnormality, an observation we failed to replicate in the present study. An important factor to consider is, whilst not standard care early in the COVID-19 pandemic, patients in the present study requiring supplemental oxygen would have received treatment with dexamethasone unless contraindicated [17]. Consistent with our previous observations follow-up LDH was significantly higher in those with persistent CXR abnormality and further baseline LDH significantly correlated with follow-up LDH concentrations. It may be that delayed normalisation of LDH reflects ongoing lung tissue inflammation/injury [18]. A hypothesis that would require testing in a formal mechanistic study.

The moderate performance of our original 5-point score, and poor discrimination of obesity, in the present study may relate to the inherent differences between the two study cohorts including, the introduction of standard of care COVID-19 therapies, the emergence of SARS CoV-2 variants [19], and the COVID-19 vaccination programme in the UK [20]. A further difference we noted was that our 1st wave cohort was enriched for patients admitted to level 2/3 care, reflecting clinical prioritisation in response to an emerging global pandemic, whereas the present study consisted of all hospitalised patients attending a dedicated follow-up service.

This study has limitations. In our established methodology, chest radiographs were not dual reported, hence it is not possible to assess concordance between assessors. However, we believe this increases the real-world applicability of our observations. Second, the score was designed to identify those at risk of persistent CXR abnormality, with its negative predictive value then appreciated. This construct may increase the scores clinical utility by reducing the impact of any missing clinical data. As once a threshold is reached further data does not alter score outcome (e.g., follow-up not required vs. follow-up recommended). A further limitation is that, due to lack of available data, it was not possible to control for any impact of patient vaccination status, SARS CoV-2 variants, or use of targeted COVID-19 therapies on the results. However, despite the differences in the disease and available treatments between cohorts, our revised score performed strongly in both. Within this study design we cannot definitively predict whether patients with CXR abnormality at 12-weeks will develop lasting radiological pathology. Although, in the present analysis we identify a group of patients in which routine radiological follow-up may not be required. Future research should focus on investigating the utility of this, or other identified predictive models, on persistent symptoms following COVID-19 infection.

In conclusion we identify, in two independent cohorts of patients hospitalised with COVID-19, a 5-point scoring system which accurately predicts patients at risk of persistent CXR abnormality at the 12-week timepoint. This score could be used as a tool to aid clinicians in triaging a group of patients that do not require routine radiological follow-up. External validation of these observations is required.

## Electronic supplementary material

Below is the link to the electronic supplementary material.

Supplementary Material 1

## Data Availability

All data generated or analysed during this study are included in this published article [and its supplementary information files].

## List of abbreviations

95%CI: 95% Confidence interval
AUROC: area under the receiver operating characteristic curve
BMI: body mass index (kg.m^− 2^)
COVID-19: coronavirus disease 2019
CT: computerised tomography
CXR: chest radiograph
HR: Hazard Ratio
IQR: interquartile range
LDH: Lactate dehydrogenase
Level 2: High dependency facility
Level 3: Intensive care facility
LOS: Length of hospital stay
SARS CoV-2: Severe acute respiratory syndrome coronavirus 2
PCR: Polymerase chain reaction
UHSFT: University Hospitals Southampton NHS Foundation Trust
