# Supplementary material for: Predicting the risk of chest radiograph abnormality 12-weeks post hospitalisation with SARS CoV-2 PCR confirmed COVID-19
Source: Respir Res. 2022 Oct 31;23:297. doi: 10.1186/s12931-022-02217-0 (PMC9620600; doi:10.1186/s12931-022-02217-0)
Supplement: Supplementary file 1 — Supplementary Material 1 [file 12931_2022_2217_MOESM1_ESM.docx]

**Supplemental File**

**Predicting the risk of chest radiograph abnormality 12-weeks post-hospitalisation with SARS CoV-2 PCR confirmed COVID-19.**

**Authors** TJM Wallis^1^, B Welham^1^, A Kong^1^, T Morelli^1^, A Azim^1^, J Horno^2^, M Wilkinson^2^, H Burke^1^, A Freeman^1^, TMA Wilkinson^1^, MG Jones^1^ and BG Marshall^1^

**Affiliations**

1. Department of Respiratory Medicine and Southampton NIHR Biomedical Research Centre, University Hospital Southampton and School of Clinical and Experimental Sciences, Faculty of Medicine, University of Southampton, Southampton UK
2. Department of Respiratory Medicine, University Hospital Southampton, Southampton UK

**Supplemental Table 1**

| **Variable** | **Present study**  **(2^nd^ Wave Cohort) n=182** | **Previous study^**  **(1^st^ Wave Cohort) n=101** | **p value** |
| --- | --- | --- | --- |
| **Sex (male)** | 54% (n=98) | 54% (n=54) | 0.910 |
| **Age (years)** | 58.0 (47-67) | 53.0 (45-63) | 0.069 |
| **Hospital length of Stay (days)** | 9.0 (8-13) | 9 (5-17.5) | 0.447 |
| **Follow-up CXR interval (days)** | 80 (74-86) | 82 (77-86) | 0.112 |
| **Level 2 or 3 Care** | 31% (n=57) | 48.5% (n=49) | 0.005** |
| **BAME** | 27% (n=47) | 35% (n=35) | 0.126 |
| **Obesity (BMI>30 kg/m^2^)** | 44% (n=80) | 28% (n=28) | 0.026* |
| **Ever smoker** | 44% (n=80) | 35% (n=35) | 0.126 |
| **Diabetes Melitus (all types)** | 20% (n=37) | 18% (n=19) | 0.726 |
| **Hypertension** | 23% (n=42) | 35% (n=36) | 0.027 |

**Supplemental Table 1 Comparison of baseline demographics of the present study cohort (2^nd^ wave cohort n=182) to our previous study cohort (1^st^ wave cohort n=101).** Values presented as median (Interquartile range) for continuous variables and percentage (n) for categorical variables. CXR-chest radiograph, Level 2 - High Dependency Facility, Level 3-Intensive Care Facility , BAME -Black, Asian and Minority Ethnic. BMI body mass index (kg/m^2^), * p<0.05 **p<0.01. Comparison of data for complete resolution versus persistent CXR abnormality assessed using the Mann-Whitney U Test or Chi Squared test as appropriate . ^Previous study results published in ref (1).

**Supplemental Figure 1**

**Supplemental Figure 1 Chest radiographs of two patients with severe COVID-19 pneumonia demonstrating bilateral pulmonary infiltrates.** A) Patient A, B) Patient B. Patient B currently receiving invasive mechanical ventilation (Level 3 care). Electrocardiogram (ECG) leads, right-sided internal jugular central venous catheter, endotracheal tube, and nasogastric tube also visible on the image. Consent for use of images obtained through the REACT study (REC: 20/SC/0138).

**References**

1. Wallis TJM, Heiden E, Horno J, Welham B, Burke H, Freeman A, et al. Risk factors for persistent abnormality on chest radiographs at 12-weeks post hospitalisation with PCR confirmed COVID-19. Respiratory research. 2021;22(1):157.
